# Yellow Nail Syndrome and Nail Lichen Planus may be Induced by a Common Culprit. Focus on Dental Restorative Substances

**DOI:** 10.3389/fmed.2014.00046

**Published:** 2014-12-02

**Authors:** Léon-Robert Baran

**Affiliations:** ^1^Nail Disease Center, Cannes, France; ^2^Gustave Roussy Cancer Institute Villejuif, Paris, France; ^3^University of Franche-Comté, Besançon, France

**Keywords:** yellow nail syndrome, lichen planus, ental restorative materials, etiologies, discoloration

## Abstract

Different clinical appearances such as Yellow nail syndrome and Lichen planus or lichenoid reactions can originate from close or identical etiologies. They may result from dental restorative materials or metal allergy. Interestingly, the nail sometimes returns to its normal condition, months after the withdrawal of the offending agents.

## Introduction

Yellow nail syndrome (YNS) is characterized by nail changes, respiratory disorders, and lymphedema (1). The yellow nails are unsightly, discolored and hard, show transverse over-curvature, and are slow growing. Paronychia and onycholysis may be observed. Nail abnormalities may be the only pattern of this syndrome.

The diagnosis of lichen planus of the nails (NLP) may be readily made when it exists together with typical cutaneous or mucous membrane disease. However, in those cases (about 10%) in which the nail is involved, a definitive diagnosis must be rendered prior to instituting therapy. NLP alone is rare (1–2%) and presents a difficult diagnostic challenge. The clinical signs of NLP are dependent on the anatomic site of the nail unit that is involved in the process. Matrix disease may vary from small atrophic foci to extensive destruction of the entire fabric of the nail. When the former change affects the proximal matrix, longitudinal grooves alternating with normal nail plate areas appear clinically as onychorrhexis. Should the entire length of the matrix be involved in the same process, actual splits occur. When diffuse atrophy of the matrix occurs and becomes functionally shorter, a thinner nail plate results. The change is usually permanent. Severe destruction of the matrix with subsequent scar formation gives rise to the specific clinical sign of lichen planus known as pterygium. Taking into account the risk of permanent dystrophy, nail biopsy is mandatory before any treatment.

Lichen planus of the nails may have features in common with that of the skin. These include hyperkeratosis, hypergranulosis, irregular epidermal hyperplasia, necrotic keratinocytes (Civatte bodies, hyaline bodies), vacuolar alteration, dense lichenoid lymphohistiocytic infiltrate with melanophages in the upper dermis that obscure the dermoepidermal interface, and coarse collagen bundles in a thickened papillary dermis.

The purpose of this paper is to demonstrate that common etiologies may be responsible for diseases at first glance far from being close such as YNS and NLP.

Berglund and Carlmark (2) examined nail clippings from patients with one or more features of YNS. “Their nails were analyzed by energy dispersive X-ray fluorescence: titanium (Ti) was regularly found in fingernails in patients but not in control subjects. Visible nail changes were present in only half of the patients. The diagnosis of YNS remains strictly clinical. Nail changes are the most common presentation (89%) followed by lymphedema (80%) and pleural effusion (63%). Only 30% of all cases have presented with the classical triad (3). The dominant source of Ti ons was Ti implants in the teeth or elsewhere. The Ti ions were released through the galvanic action of dental gold or amalgam or through the oxidative action of fluorides. Stopping the galvanic release of Ti ions or canceling exposure to Ti dioxide led to recovery. In one patient with a Ti implant, the symptoms recurred after renewed exposure to Ti.”

Four men and four women had symptoms of YNS after exposure to Ti dioxide in drug tablets or confectionary (chewing gum). In fact, the dominant cause of yellow nails in this article was the galvanic interaction between gold in the teeth and Ti implants.

Several publications mention the exposure to drugs (that actually contain Ti dioxide) preceding the development of yellow nails and also the return to normal conditions months after withdrawal of the drugs (4–6).

Fluorides ions are the only aggressive ions which provide a protective oxide layer for Ti and Ti alloys. Since hygiene products like tooth pastes and prophylactic gels contain fluorides ions, Reclaru and Meyer (7) have evaluated the effect of fluoride ions on Ti and dental alloys used, for example dental implants (a surgical component that interfaces with the bone of the jaw or skull to support a dental prosthesis) and superstructures (portion of a dental implant). “In confined areas where fluoride ions are present, Ti and the dental alloys tested undergo a corrosive process in the crevices and pits as soon as the pH drops below 3.5,” which is observed in any case of dental infection such as lesions of endodontic origin.

Although metal allergy has been considered to be one of the precipitating factors in LP, there are only few reports that have shown a clinical association between metal allergy and NLP. In the study of Nishizawa et al. (8), “the prevalence of positive metal patch test (PT) results were higher in NLP as compared with oral lichen planus (OLP). In addition, 60% of these NLP patients (6 of 10 cases) showed clinical improvement after either removal of the dental materials or systemic disodium cromoglycate therapy. Positive Patch testing (PT) to metals, such as Cr, Ni and Au, done in patients were detected in both the dental fillings/braces and in the nail lesions in some of the authors’s NLP patients. These data suggest that metals released from dental materials contributed to the development of NLP via systemic absorption and subsequent deposition in the nail tissues.”

Kato et al. (9) have reported on a patient with lichen planus of the buccal mucosa (OLP), the nails, and the skin were caused by mercury allergy. “Since 1998, a 61-year-old man had felt stinging on his buccal mucosa when he ate or brushed his teeth and all of his nails became rough and thin in June 2000. All the lesions biopsied showed a dense band-like lymphocytic infiltrate in the upper dermis and liquefactive degeneration of the basal layer. Patch testing (PT) showed positive reactions to the toothpaste that he used sodium *N*-lauroyl sarcosinate 0.5% aq, mercury 0.05% pet, ammoniated mercuric chloride 1.0% pet, and thimerosal 0.1% pet. Mercury was used as a component of his dental fillings. When these were replaced by the hard resin jacket crowns, the mucosal, and nail symptoms gradually improved.”

A similar case of allergy due to mercury amalgam has been presented by Higashi (10) where a patient developed a systemic LP associated with NLP restricted to the toes.

Yokoseki et al. (11) examined “a 51-year-old man with 20-nail dystrophy produced by lichen planus, which was histologically confirmed by a longitudinal biopsy which included the nail matrix. All the nails had rough surfaces and were atrophied distally and the proximal nail folds were reddish and swollen. The patient complained of severe itching in these regions.” The authors carried out patch-tests, which revealed positive reactions to both nickel and gold. An electromicroanalysis revealed that gold but no nickel had been used as a component of the dental fillings. After these fillings were replaced by hard resin, jacket crowns, the nail symptoms dramatically improved.

Takeuchi et al. (12) have observed the case of a 57-year-old man who had deformities of all 10 fingernails for 18 months before presentation and deformities of all 10 toenails, for the previous 6 months. The surface of the nails was rough, with excessive longitudinal ridging. The base of the nails was slightly hypertrophic, but their tip was atrophic. “All proximal nail folds were reddish and swollen. The patient complained of severe itching of the region of the fingernails and fingernail folds. A longitudinal nail biopsy including the nail matrix revealed the typical histology of lichen planus. There were also reticular-shaped, milky-white macules, and erythemas with pigmentations on both buccal mucous membranes (11). Histological analysis showed lichen planus intermingled with eosinophils. Immunological blood analysis revealed elevated CD4^+^ T cells and CD4/CD8 ratio. The patient worked as a tinsmith for over 30 years and had had dental metal on several teeth for approximately 20 years. The metal serie patch test revealed positive reactions to chromate and tin. Treatment with systemic steroids was quite effective in treating the nail lesions.”

## Commentary

Dental restorative substances are not the only ones responsible for either YNS or NLP.

Boone et al.’s (13) case report of “a 27-year-old woman who sought medical advice for purple papular confluent lesions of the soles and the lateral border of both feet, which appeared isolated. They had started 4 years previously and were associated with dystrophic nails, which were more prominent on the toes than on the fingers. The patient was also complaining about painful oral and genital erosions with some synechia in the latter area. A biopsy confirmed the clinical diagnosis of lichen planus. The biological examination results (not specified) were normal. Ciclosporine A was prescribed and after 1 month, there was a dramatic improvement. As the patient had been working for 4 years in the photographic industry (Canon and Agfa Gevaert), PT was performed. In the photographic chemical serie, it revealed a positive lichenoid reaction after 72 h to metol (4-methyl aminophenol) and phenidone (1-phenyl-3pyrazolidinone) and, in the metal ions serie, reactions to gold sodium thiosulfate. Careful consideration was given to the workplace survey of the patient and showed that all day long she had contact with metol, phenidone, and gold sodium thiosulfate. After 6 months treatment, the patient, completely cured, stopped ciclosporine.” Consequently, it is tempting to consider that color-film developers might have exhibited a lichen planus induced by contact to the mentioned agents.

It seems also logical to establish a connection between hydroxylamine, both as a sensitizer and as an irritant to Boone et al.’s observation (13). Hydroxylamine may produce onycholysis and/or paronychia (14, 15). Moreover, it has been widely used in color photograph processing, the chemical industry (oximes synthesis), the pharmaceutical industry (bactericide, fungicide, antialgal), and in the manufacture of rubber and plastic compounds, cosmetics, and soap.

More unusual are the three cases of Sodhi et al. (16) “who developed a contact dermatitis of periorbital skin and NLP following the use of topical brimonidine (0.2% twice a day) for primary open angle glaucoma. These side-effects slowly disappeared on discontinuing the drug but reappeared on reintroducing topical formulation.”

## Discussion

Different remarks deserve some consideration. Chemically induced LP has been described with amalgam such as Hg, products containing thiol or releasing SH (D-penicillamine that may also induce YNS in addition to captopril, gold sodium thiomalate) and external exposure to a chemical agent, which was not a drug such as color-film development materials.

Some authorities have tried to separate lichenoid reactions from true lichen planus. Unfortunately, it is usually impossible to distinguish these conditions with certainty, even histologically and we should accept that there is a wide etiologic range that has to be recognized. Although metal allergy has been considered to be one of the precipitating factors in LP, there are only few reports that have shown a clinical association between metal allergy and NLP.

Many workers in color-film development have presented with lichen planus or lichenoid reactions (17–22) but unexpectedly only one case was associated with “nail dystrophy” affecting all the digits (13).

Finally, the enigmatic yellow discoloration observed in some cases of isolated NLP (23–26) may well be considered as a clinical bridge between LP and YNS. However, above all, we have tried to demonstrate that a common culprit may be a link for these quite different diseases.

## Conflict of Interest Statement

The author declares that the research was conducted in the absence of any commercial or financial relationships that could be construed as a potential conflict of interest.
